# Development of a Regional Nursing Research Partnership for Academic and Practice Collaborations

**DOI:** 10.1155/2013/473864

**Published:** 2013-07-28

**Authors:** Heather L. Tubbs-Cooley, Donna S. Martsolf, Rita H. Pickler, Caroline F. Morrison, Cassie E. Wardlaw

**Affiliations:** ^1^Cincinnati Children's Hospital Medical Center, 3333 Burnet Avenue, MLC 11016, Cincinnati, OH 45229, USA; ^2^College of Nursing, University of Cincinnati, 249 Procter Hall, Cincinnati, OH 45221, USA

## Abstract

*Background.* Collaborative nursing research across academic and practice settings is imperative to generate knowledge to improve patient care. Models of academic/practice partnerships for nursing research are lacking. This paper reports data collected before and during a one-day retreat for nurse researchers and administrators from local universities and health care organizations designed to establish a regional nursing research partnership. *Methods.* Quantitative and qualitative methods were used to address the study aims: (1) to assess research involvement and institutional research resources; (2) to assess interest in and concerns regarding cross-institutional collaborations; and (3) to describe perceptions of the purpose of a partnership and resources needed to ensure success. *Results.* Participants (*n* = 49) had differing perceptions of accessibility to resources; participants in practice settings reported less accessibility to resources, notably grant development, informatics, and research assistant support. Participants were interested in collaboration although concerns about conflict of interest were expressed. Four themes related to partnering were identified: harnessing our nursing voice and identity; developing as researchers; staying connected; and positioning for a collaborative project. *Conclusion.* Academic-practice research collaborations will become increasingly important with health care system changes. Strategies to develop and sustain productive partnerships should be supported.

## 1. Background

Collaborative nursing research across academic and practice settings is essential for the advancement of science that generates new discoveries and determines effective approaches for research translation and implementation. Multiple national and international studies highlight benefits of academic-practice research partnerships such as improving the validity and effectiveness of interventions via varied stakeholder perspectives [1–6], efficiency gains in personnel and infrastructure costs owing to shared resources [7], and improved patient outcomes resulting from implementation of evidence-based nursing care practices [8]. In spite of these benefits, models to engage academic nursing faculty and practice-based researchers and clinicians in collaborative research are limited. The Duke Translational Nursing Institute is a recent example of an academic-practice partnership between the Duke University School of Nursing and the Duke University Health System and is designed to align academic and clinical nurses in the pursuit of new knowledge and the rapid translation of knowledge into clinical nursing practice [8]. While the Duke model holds promise for large academic medical centers, additional models of nursing research partnerships are needed to extend the benefits of knowledge generation and implementation more equitably across multiple organizations within a community.

 The Cincinnati Partnership for Nursing Research (CPNR) is a nascent initiative supported by the University of Cincinnati's Clinical and Translational Science Award, local universities, and local health care organizations to promote research collaboration among academic and practice-based nurse researchers across the greater Cincinnati metropolitan area. The CPNR is a response to multiple stimuli: a growing realization among researchers that synergistic and mutually beneficial opportunities are often missed as a result of a historically isolated approach to research, a desire to access patient populations for research in various organizations, and the recognition that larger, well-coordinated studies are more likely to impact the health of the community. In October 2012, academic nursing faculty, practice-based nurse scientists, academic deans, and nurse executives were invited to an inaugural retreat to assess the current state of nursing research collaboration across the community and to initiate the development of a strategic research plan to address the pressing health needs of the local population. The retreat presented an opportunity to gather data on invitees' views of challenges and opportunities related to cross-institutional collaboration. The purpose of this paper is to report these data, which were collected using quantitative and qualitative methods according to three specific aims: (1) to assess invitees' research involvement and their access to institutional research resources; (2) to assess invitees' interest in and concerns regarding cross-institutional collaborations; and (3) to describe participants' views of the purpose of a partnership and the resources needed to ensure success.

## 2. Methods

 We used a multimethod approach to collect descriptive data related to the aims of the study. The target population for the study included academic nurse faculty, practice-based nurse researchers, nurse executives, research-focused doctoral students, and representatives of community health organizations. Approximately 110 individuals distributed among these categories were invited to attend the CPNR retreat based on their role in their organization as either conducting or supporting nursing research. The invitees constituted the purposive sample for the quantitative survey data, while the 64 attendees comprised the purposive sample for the qualitative data collection.

### 2.1. Quantitative Survey Data Collection and Analysis

Prior to the retreat, a 31-item web-based survey was constructed to anonymously query invitees about characteristics of their current position, research experiences, access to institutional research resources, and interest in collaborative research. All participants progressed through the survey in a similar manner until designating oneself as primarily a researcher or an administrator, at which time they were presented with separate sets of questions relevant to their respective role. Upon institutional review board review and approval, we sent an initial email solicitation to all invitees with explanation of the survey and an embedded web link. We followed the initial solicitation with two additional email solicitations, and we received completed surveys from 49 invitees (45% response rate). Completion of the survey constituted informed consent.

Data analysis for the survey consisted primarily of descriptive summary statistics including measures of central tendency (mean, median) and dispersion (standard deviation, range). Fisher's exact tests were used to assess differences in responses among participants based in different settings. All quantitative analyses were conducted using Stata 11.0 (StataCorp, College Station, TX, USA), and results were considered statistically significant at *P* < 0.05.

### 2.2. Qualitative Data Collection and Analysis

There were 70 participants at the retreat; additional demographic data were not collected from attendees. The “world café method,” a format for hosting large group dialogue, was used to gather data from retreat participants [9]. The café was organized into seven tables, each with an assigned question as listed in Box 1. Participants were assigned a table rotation such that each participant was at each table and each of the seven rotations were mixed so that no group of participants ever followed the same rotation. Each rotation was 15 minutes in duration; table facilitators (faculty) and scribes (doctoral students) took notes at each rotation. At the start of each rotation, facilitators gave a brief synopsis of the discussions that had occurred at that table with a goal of building on each rotation's dialogue. At the end of the seven rotations, we had an open discussion involving all the participants and gained agreement with the major table themes. Data from the world café dialogue were more thoroughly analyzed by the authors, who read the content of these café notes independently and then met as a research team to discuss the data and reach consensus about how the data clustered.

## 3. Results

### 3.1. Quantitative Results

 Characteristics of the 49 survey participants are shown in Table 1. Slightly more than half were from practice-based settings, while 6.5% identified themselves as affiliated with community organizations. Participants were evenly distributed according to their primary role as either a researcher or as a person supporting the conduct of others' research, such as a chief nursing officer. Approximately half had an active research study as either a principal or coinvestigator, and several of these studies were externally funded through federal and foundation research grants. Although nearly half of survey participants reported membership in the University of Cincinnati Center for Clinical and Translational Science and Training (CCTST) funded by a Clinical and Translational Science Award (CTSA) from the National Institutes of Health, approximately 44% were unaware of the CCTST and its research mission and available resources.

 Survey participants in academic and practice settings had differing perceptions of accessibility to research resources and these differences were often significant (Table 2). Those in practice settings reported less accessibility to resources overall and significantly less accessibility to grant development support, informatics support, and research assistant support. There were no significant differences between academic and practice-based participants' levels of interest in various types of collaborative work including development of extramural funding proposals, joint publications, evidence-based practice projects, and staff nursing research to meet Magnet Recognition Program objectives (Table 3). 

 We asked participants at the close of the survey to report the degree to which they were concerned about conflicts of interest when collaborating on projects with researchers in other organizations.  Table 4 highlights these responses; most participants reported some degree of concern about conflicts of interest and concern was not significantly different between academic and practice-based participants. Only 28.2% of participants reported no concern at all.

### 3.2. Qualitative Results

The research team agreed that the all dialogue data could be placed into one of 9 categories: visibility/identity; support/resources; skills; partnerships/collaboration; human capital; interest/motivation; community/professional organizations; communication/information; and pressing health concerns. The research team further examined the categories for overlap. This examination resulted in the team identifying four themes that captured the dominant thoughts of participants about the nature and purpose of partnerships and what was needed and available to make partnerships a reality. The four themes were harnessing our nursing voice and identity; developing as researchers; staying connected; and positioning for a collaborative project. This analysis provides an understanding of what the retreat participants perceived should be the nature and purpose of a nursing research partnership.

The first theme was *harnessing our nursing voice and identity*. The 70 individuals at the retreat were meeting all together for the first time. This theme reflected the participants' excitement about coming together as a group who could collectively make nursing's contribution to research more visible. Participants indicated that harnessing the nursing voice and identity was made possible because of the composition of the group that included nurse scientists, clinicians, administrators, students, policy makers, and community members from a number of institutions. Representatives from three local, health-related foundations and councils offered their assistance to increase the visibility of the CPNR and to facilitate collaboration beyond nursing. At the conclusion of the world café discussion, the group identified nine individuals representing varied constituencies to meet with the directors of the CCTST. One of the purposes of the meeting was to articulate the participants' belief that, as nurses, we are uniquely positioned to initiate and lead studies examining the wide range of health-related issues identified at the retreat and described later.

The second theme was *developing as researchers*. Many participants indicated that they needed expanded knowledge, skills, and resources to further develop their research capabilities. For example, participants were able to clearly articulate important research areas. However, many participants noted that they lacked the skill needed to translate these ideas into researchable questions, including determining appropriate research methods. Participants were hopeful that mentoring could be provided by more experienced researchers in the group. They also wanted to incorporate undergraduate and doctoral students into the group both to assist with the research process and to be mentored in the research process. Very few participants indicated that they had prior knowledge about the services of the CCTST. Information was provided about the CCTST and almost all participants indicated a commitment to join and to use the research support services. Participants used the world café format as a forum to articulate to the nursing administrators in the group their desire for workplace support to develop as researchers. Participants indicated that workplace support includes dedicated time for research and funding for further education as well as for research projects. 

The third theme was *staying connected*. Participants were clear that they wanted to continue to work towards an effective partnership involving area nurse researchers and administrators. They saw effective partnership as learning what research CPNR nurses were conducting and exploring future opportunities to work across institutions on shared problems. At the conclusion of the world café dialogue, the group was committed to using technology to develop communication avenues and the director of a local health council volunteered to lead a group to develop a CPNR website. 

The fourth theme, *positioning for a collaborative project*, was developed from the ninth category of pressing health concerns in the community. This category contained a lengthy list of health, health system, and community issues. The research team examined the health concerns list further and identified six areas of pressing concerns: care access and health disparities; care continuity; safety; health promotion/disease prevention; chronic illness/multiple illness conditions; and environmental and cultural issues. The participants thought that these broad topical areas reflected the health phenomena that have been the concern of nurse researchers for decades. Although the participants acknowledged that as a group we are not yet ready to pursue a collaborative research project, participants also agreed that care continuity was likely the most pressing current concern encompassing the issues of patient discharge, transition to home or other health care facility, and readmission risk. It was generally agreed that a collaborative study using shared data from electronic health records and other data sources could be undertaken by group members to help inform future best practices. 

## 4. Discussion

 In our descriptive analysis of survey data regarding collaborative research across researchers and organizations within a large metropolitan area, we found significant differences in perceptions of access to research resources between participants in academic and practice-based settings. We also found areas of likely research collaboration but not without concerns about conflicts of interest. Our qualitative analysis of retreat dialogue resulted in the identification of four themes: harnessing our nursing voice and identity to increase the visibility of nurses' contributions to science and research teams; developing as researchers to increase scholarly capability; staying connected for networking and awareness; and positioning for a collaborative project that is both beneficial to the community and competitive for extramural funding.

 Access to research resources is a consistently reported concern for nurses and for practice-based nurses in particular [9–11]. Although the number of clinically based nurse researchers continues to rise, it is not necessarily the case that adequate resources are available for the conduct of meaningful, generalizable research. Moreover, as the retreat participants noted, many practice-based nurses with research responsibilities perceive themselves to be inadequately prepared to manage the challenges of conducting rigorous research. In particular, lack of preparation in and support for methods development, statistical analysis, and dissemination work, including writing manuscripts, is a perceived barrier to research work. We believe this is one area where academic-practice partnerships may be productive, as most academic faculty have skills and resources that can be shared with practice-based research colleagues. Additionally, we have sought to collaborate with our local CTSA to work on the development of resources that might be made available to both academic and practice-based nurses, such as statistical and methodological consultations and research training opportunities.

A number of survey participants expressed concern about conflicts of interest in collaborative research. Our survey did not allow for identifying the root of these concerns. Thus, it is not clear if the concerns have to do with financial matters, sharing of proprietary information, loss of control over institutional information and resources, or other issues. However, the fact that conflicts are a concern has prompted us to consider a future meeting to explicate conflicts and come to mutual agreements regarding boundaries and ethics in research collaborations. 

The sustainability and benefits of research partnerships are well documented [7]. For example, a collaborative partnership between a university-based school of nursing and a tertiary care hospital has yielded benefits for more than twenty years according to Horns and colleagues, providing an opportunity to creatively address a shortage of new nurses, a shortage of advanced practice nurses, and the advancement of clinical nursing research [12]. Future work of the CPNR will include setting directions for research, developing an ethical framework for collaboration, and developing and tracking outcomes associated with cross-institutional partnerships. 

Of particular interest to us was the identification of areas where nurse researchers can collaborate to improve health outcomes in the community. McInnes et al. describe clinical networks as a way to improve health outcomes and processes based on perceived local need, and five key factors were identified as important conditions for establishing a successful clinical network: building relationships; adequate resources; ability to implement and evaluate initiatives; effective leadership; and strategic plans that are evidence based [13]. We consider the CPNR to be an emerging clinical research network to conduct research that benefits the community. In our situation, there is a spectrum of research interest from small research and implementation projects to robust multisite studies submitted for federal research funding.

## 5. Limitations

The target population for this study was clinical and academic nurse researchers and nurse executives. We solicited participants from individuals who met the inclusion criteria and received an invitation to the retreat, and it is possible that the data and findings may not reflect the views of researchers/executives who did not participate in the event. We did not define the term “conflict of interest” for participants and can only speculate on how participants defined it when completing the questionnaire. Lastly, our data reflect views in in one community and additional research in other locales would be helpful to confirm the findings presented in this paper. 

## 6. Conclusions

Academic-practice research collaborations will become increasingly important as health care institutions work to adopt recommendations and standards outlined in the Institute of Medicine's 2012 report on learning health care systems [14]. Providing better care at lower cost will require reliable implementation of best evidence; yet best evidence for many care delivery practices is lacking. We believe that nurses in academic and practice settings with research and implementation skills are well positioned to generate new evidence, conduct comparisons of existing evidence-based care delivery approaches, and implement best practices and that the most innovative and scalable research will occur when investigators in a variety of settings work together in such endeavors. Further, we believe that the CPNR addresses the demand for academic-practice research collaborations as called for by national and international nursing organizations, and that it can serve as a model for other communities interested in building research capacity across settings.

Our retreat participants agreed that the future work of the CPNR should focus on (a) increasing the nursing voice and identity within the local research community; (b) assisting nurses to develop increasingly sophisticated research capabilities; and (c) disseminating information about current research activities of CNPR members and collaborating across institutions to develop research projects to address common problems. Specific goals of the partnership in the next year are (1) increasing the numbers of nurse members in the local CTSA organization; (2) developing an interactive website for the CPNR; (3) hosting a second retreat with a focus on ongoing research being conducted by members; and (4) expanding our membership to encompass all interested nurse researchers in our area.

## Figures and Tables

**Box 1:**
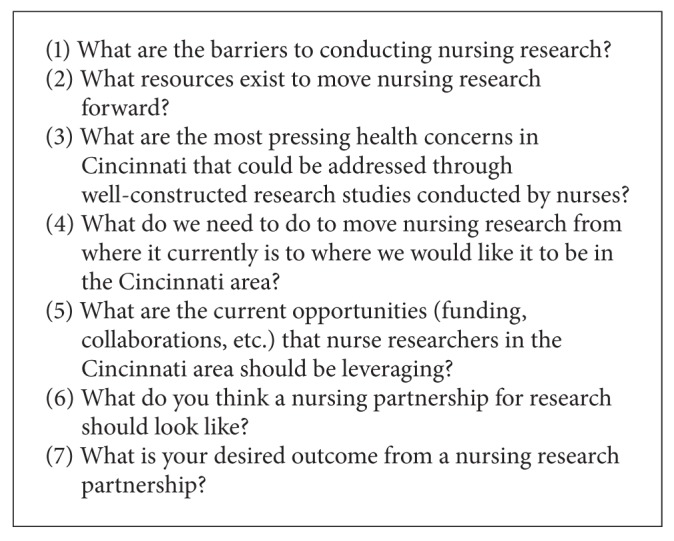
World café questions.

**Table 1:** Sample characteristics of survey participants (*n* = 49).

	% (*N*)
Primary organization	
University	19 (41.3)
Hospital/practice setting	24 (52.2)
Community organization	3 (6.5)
Primary research role	
Conduct own research	52.2 (24)
Support conduct of others' research	47.8 (22)
Interested in conducting research but need help/resources	36.7 (18)
Submitted a grant in last 5 years as PI or Co-I	51.1 (25)
Have active research study as PI or Co-I	44.9 (22)
Member of CCTST (NIH CTSA)	48.9 (22)

	Mean (SD)

Ave # peer-reviewed research pubs in last 5 years	1.7 (3.9)

**Table 2:** Accessibility of research resources by setting.

	Academic	Practice
	% (*N*)	
Administrative support		
Never accessible/mostly inaccessible	0 (0)	20.8 (5)
Sometimes accessible	20.0 (3)	8.3 (2)
Mostly accessible/always accessible	80.0 (12)	70.8 (17)
Grant development support		
Never accessible/mostly inaccessible	6.7 (1)	25.0 (6)*
Sometimes accessible	0 (0)	20.8 (5)
Mostly accessible/always accessible	93.3 (14)	54.2 (13)
Grant management support		
Never accessible/mostly inaccessible	6.7 (1)	20.8 (5)
Sometimes accessible	13.3 (2)	29.2 (7)
Mostly accessible/always accessible	80.0 (12)	50.0 (12)
Informatics support		
Never accessible/mostly inaccessible	6.7 (1)	16.7 (4)
Sometimes accessible	6.7 (1)	37.5 (9)*
Mostly accessible/always accessible	86.7 (13)	45.8 (11)
Methodological support		
Never accessible/mostly inaccessible	6.7 (1)	20.8 (5)
Sometimes accessible	6.7 (1)	29.2 (7)
Mostly accessible/always accessible	86.7 (13)	50.0 (12)
Research assistant support		
Never accessible/mostly inaccessible	13.3 (2)	45.8 (11)*
Sometimes accessible	13.3 (2)	25.0 (6)
Mostly accessible/always accessible	73.3 (11)	29.2 (7)
Statistical support		
Never accessible/mostly inaccessible	7.1 (1)	25.0 (6)
Sometimes accessible	35.7 (5)	37.5 (9)
Mostly accessible/always accessible	57.1 (8)	37.5 (9)

Note: *P < 0.05 based on Fisher's exact test.

**Table 3:** Interest in types of collaborations among academic (A) and practice (P) based participants, % (*N*).

	Staff research to meet magnet objectives		EBP projects		Developing proposals for extramural research funding		Coauthoring publications	
	A	P	A	P	A	P	A	P
Very interested	40.0 (6)	50.0 (20)	66.7 (10)	70.8 (17)	80.0 (12)	83.3 (20)	93.3 (14)	70.8 (17)
Moderately interested	26.7 (4)	15.0 (6)	6.7 (1)	0	13.3 (2)	12.5 (3)	0	16.7 (4)
Mildly interested	20.0 (3)	17.5 (7)	13.3 (2)	12.5 (3)	6.7 (1)	4.2 (1)	6.7 (1)	12.5 (3)
Not interested	13.3 (2)	17.5 (7)	13.3 (2)	16.7 (4)	0	0	0	0

**Table 4:** Degree of concern over conflicts of interest when collaborating with researchers in other organizations, % (*N*).

	Academic	Practice
Highly concerned	28.6 (4)	8.3 (2)
Moderately concerned	20.5 (8)	16.7 (4)
Mildly concerned	35.9 (14)	50.0 (12)
Not concerned	28.2 (11)	25.0 (6)
